# Supplementary material for: Trends in Pregnancy-Associated Cervical Cancer in Japan between 2012 and 2017: A Multicenter Survey
Source: Cancers (Basel). 2022 Jun 23;14(13):3072. doi: 10.3390/cancers14133072 (PMC9264791; doi:10.3390/cancers14133072)
Supplement: Supplementary file 1 [file cancers-14-03072-s001.zip › cancers-1752343-supplementary.pdf]

Supplementary Information

Title: Trends in pregnancy-associated cervical cancer in Japan between 2012 and 2017: a multicenter survey

Sayako Enomoto MD[1*]; Kosuke Yoshihara MD, PhD[2*]; Eiji Kondo MD, PhD[1]; Akiko Iwata MD[3]; Mamoru Tanaka MD, PhD[4]; Tsutomu Tabata MD, PhD[5]; Yoshiki Kudo MD, PhD[6]; Eiji Kondoh MD, PhD[7]; Masaki Mandai MD, PhD[8]; Takashi Sugiyama MD, PhD[9]; Aikou Okamoto MD, PhD[10]; Tsuyoshi Saito MD, PhD[11]; Takayuki Enomoto MD, PhD[2]; Tomoaki Ikeda MD, PhD[1]

1. Department of Obstetrics and Gynecology, Mie University School of Medicine, Mie.
2. Department of Obstetrics and Gynecology, Niigata University Graduate School of Medical and Dental Sciences, Niigata.
3. Department of Obstetrics and Gynecology, Yokohama City University School of Medicine, Kanagawa.
4. Department of Obstetrics and Gynecology, Keio University School of Medicine, Tokyo.
5. Department of Obstetrics and Gynecology, Tokyo Women's Medical University, Tokyo.
6. Department of Obstetrics and Gynecology, Hiroshima University Graduate School of Medicine, Hiroshima.
7. Department of Obstetrics and Gynecology, Kumamoto University School of Medicine, Kumamoto.
8. Department of Obstetrics and Gynecology, Kyoto University School of Medicine, Kyoto.
9. Department of Obstetrics and Gynecology, Ehime University School of Medicine, Ehime.
10. Department of Obstetrics and Gynecology, The Jikei University School of Medicine. Tokyo.
11. Department of Obstetrics and Gynecology, Sapporo Medical University, Sapporo.

Figure S1. Annual trends in the frequencies of cervical cancer during pregnancy and postpartum.

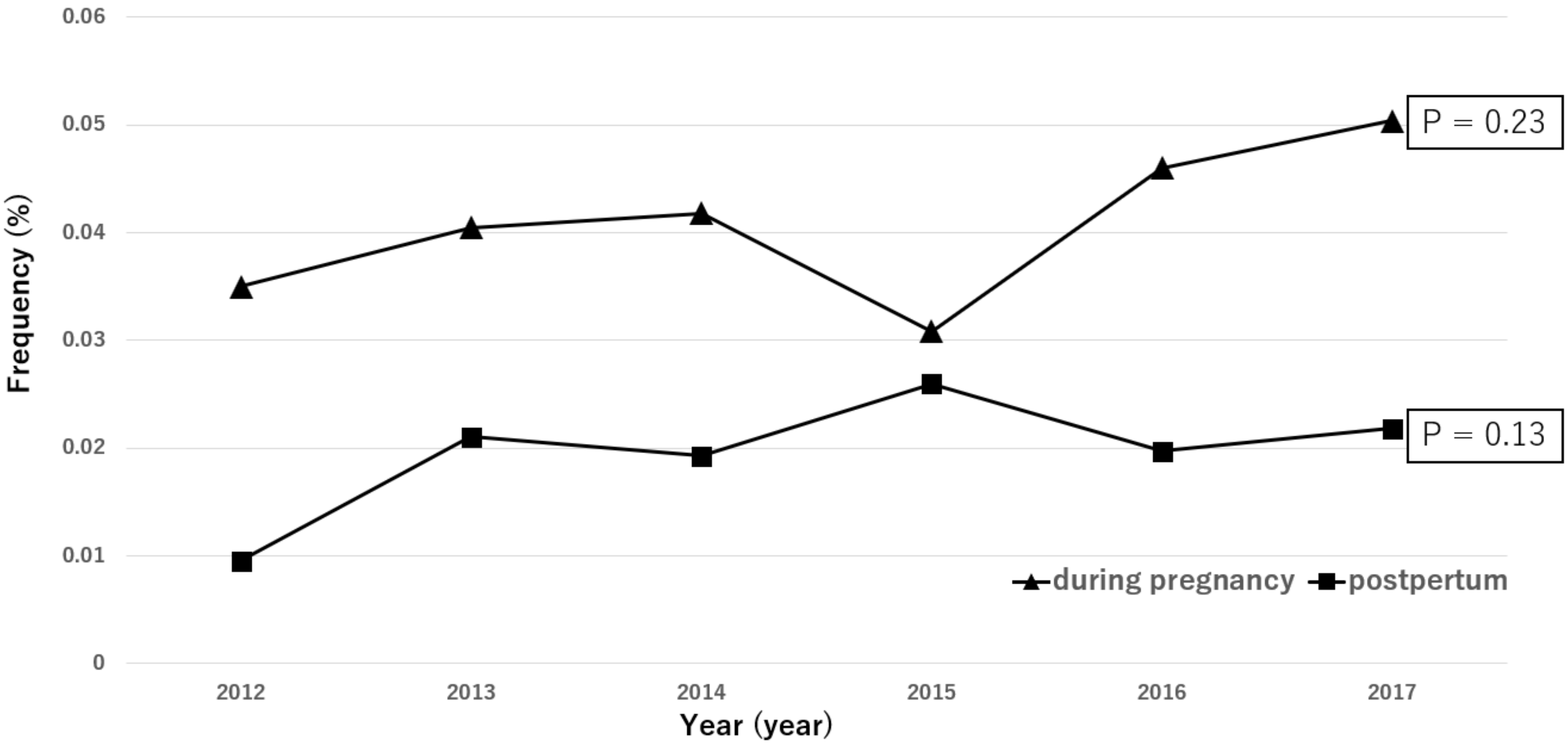

Figure S2. Time from diagnosis to delivery in the group diagnosed at ≥22 weeks of gestation

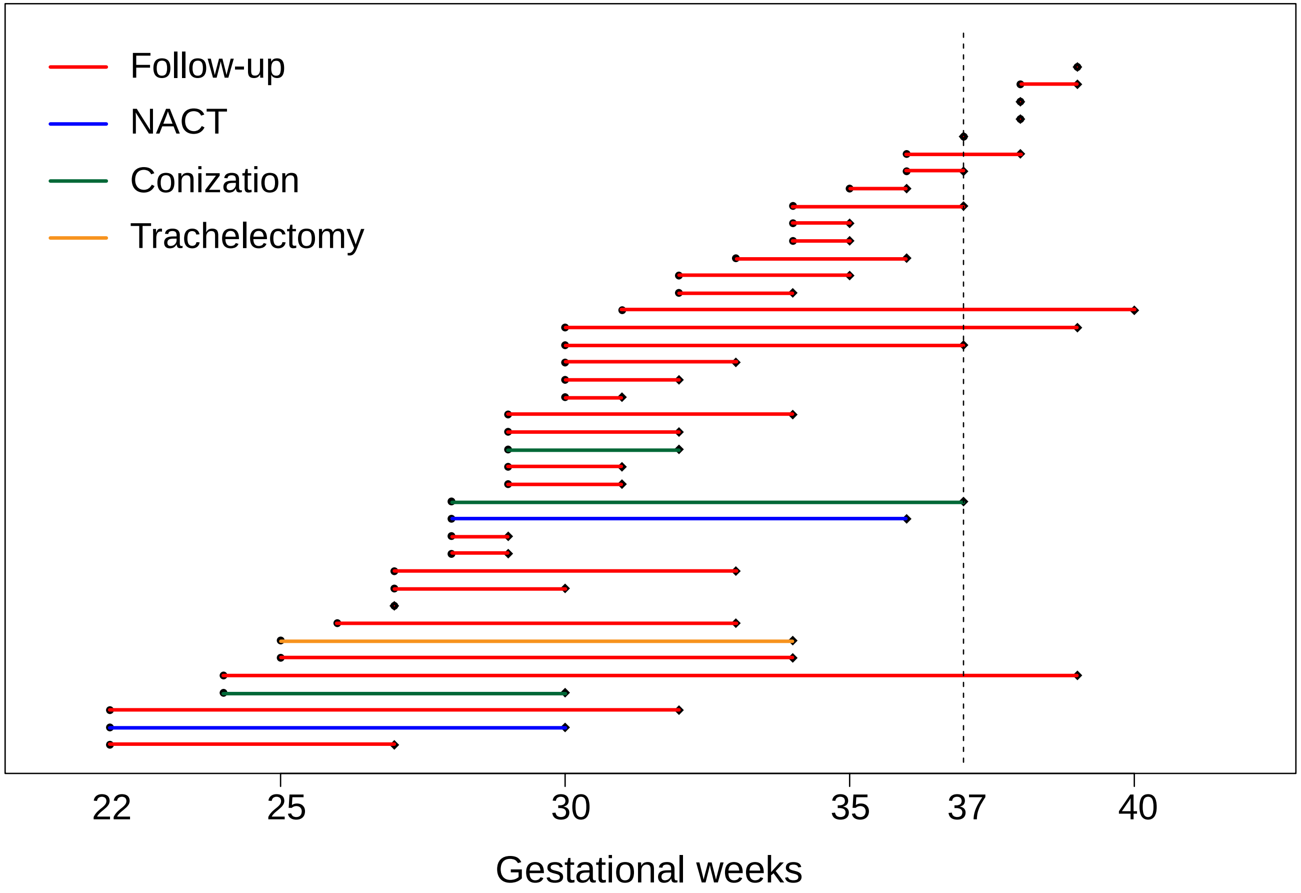

Figure S3. Outcomes during pregnancy by stage among women diagnosed at gestational age <22 weeks (n=163)

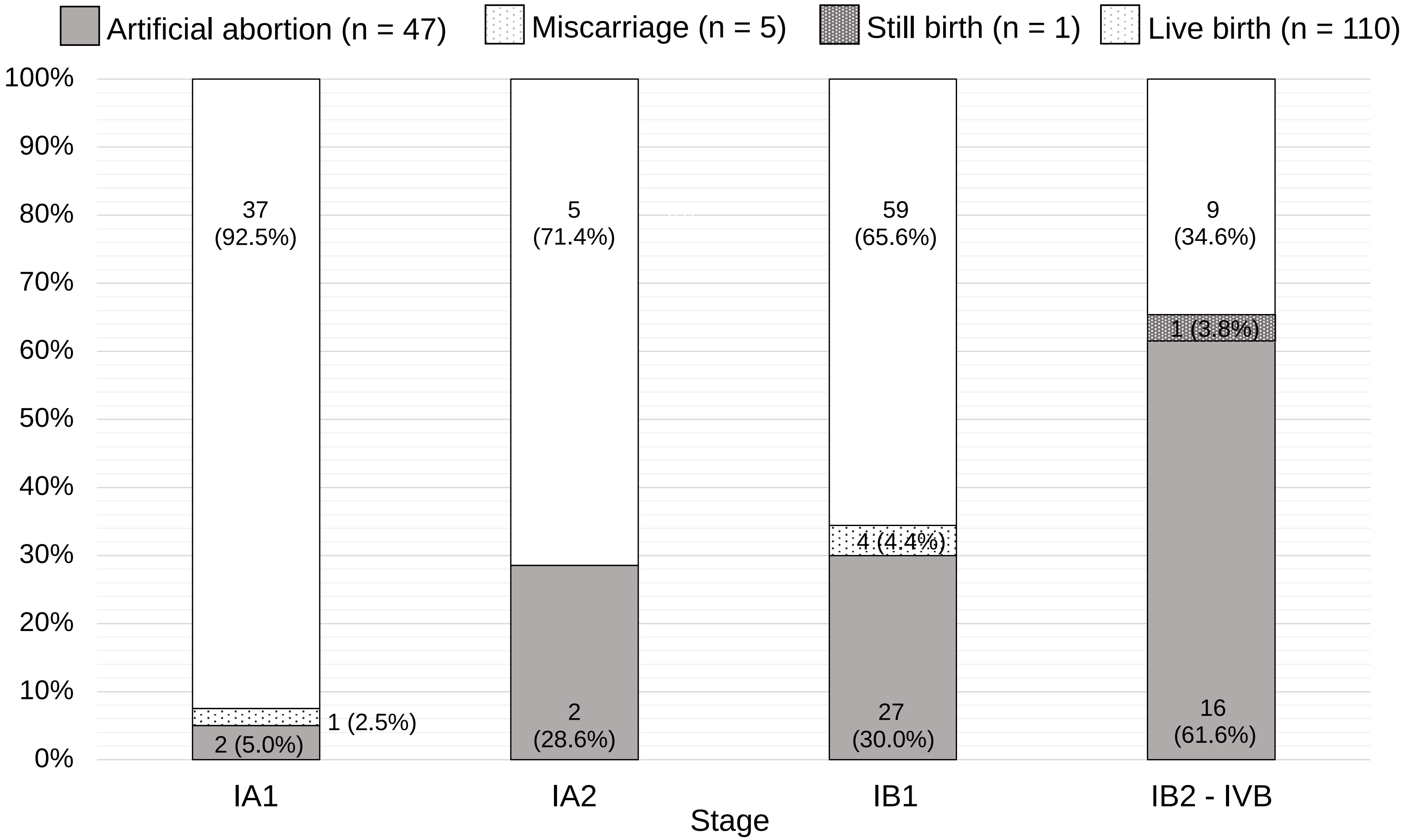

Figure S4. Pregnancy outcome of stage IA1 cases treated by conization before <22 weeks of gestation.

Duration from diagnosis to delivery (A) and the median gestational age at diagnosis and delivery (B) in stage IA1 cases who underwent conization before <22 weeks of gestation

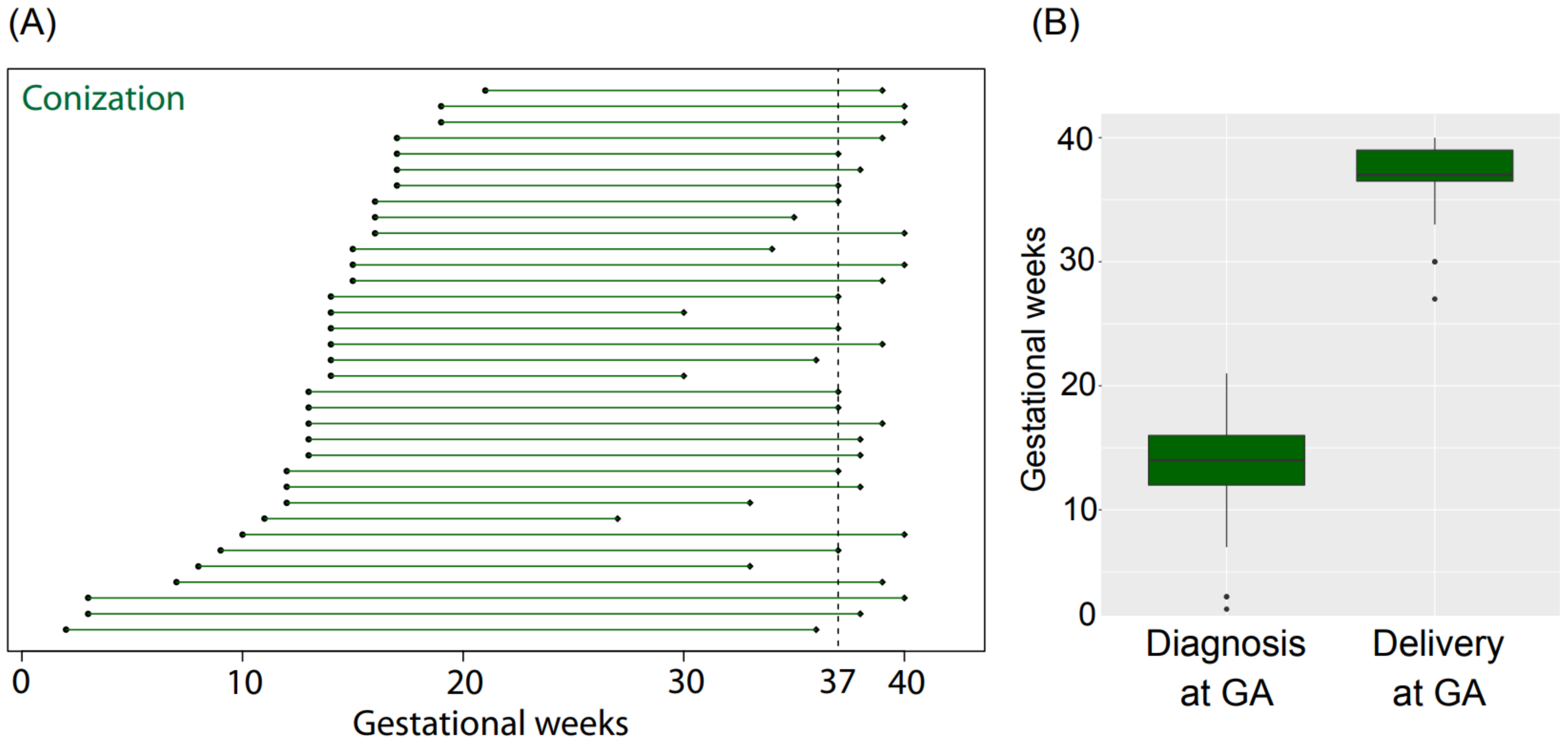

Figure S5. Subsequent treatments in the four groups

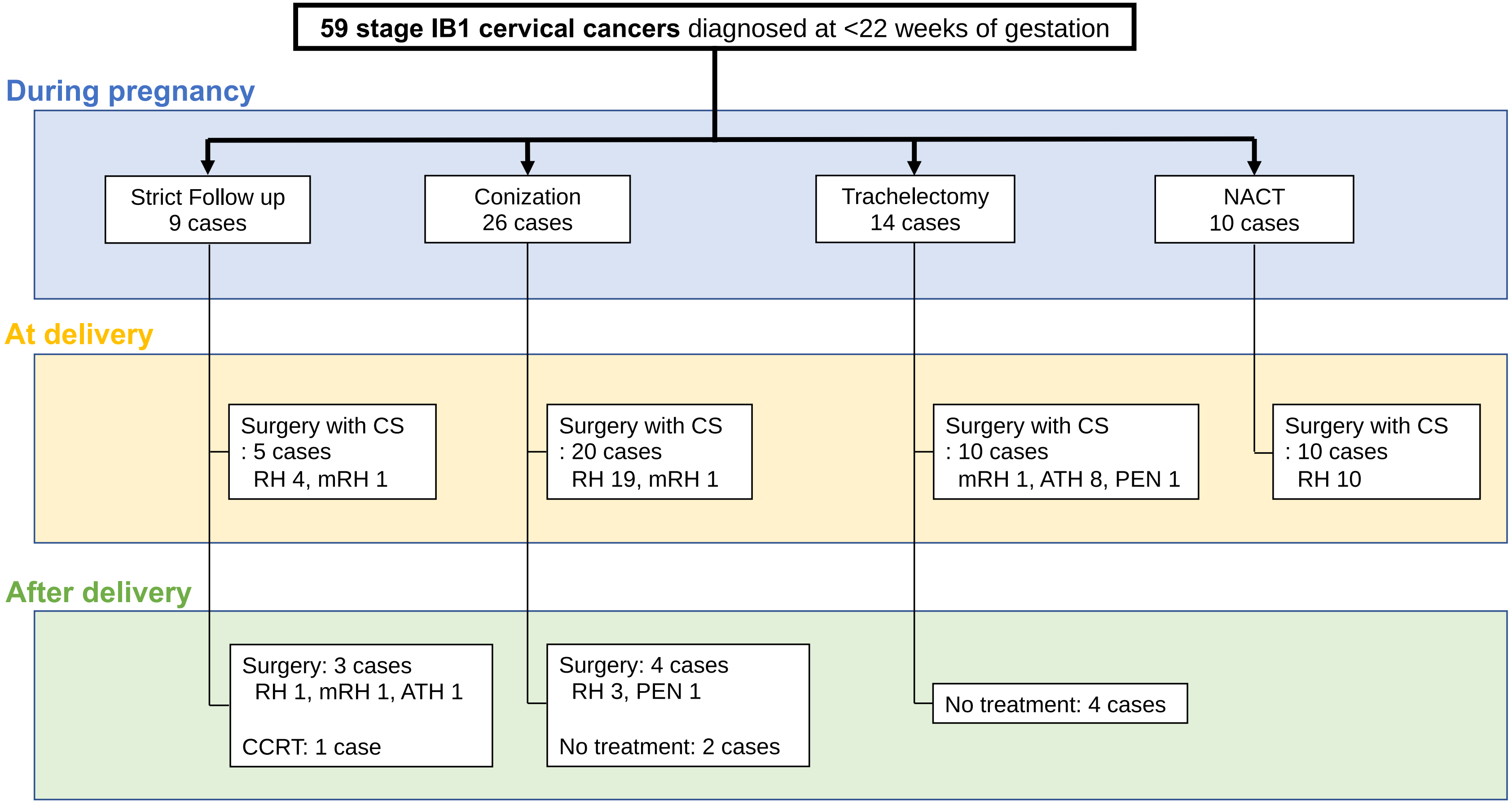

ATH: Simple hysterectomy, CCRT: concurrent chemoradiotherapy, CS: cesarian section, mRH: modified radical hysterectomy,

NACT: neoadjuvant chemotherapy, PEN: pelvic lymphadenectomy, RH: Radical hysterectomy

Table S1. Trends in treatment modalities administered during pregnancy by stage among patients diagnosed at gestational age ≥22 weeks (n=40)

**Treatment modality**	**IA1 (n=3)**	**IA2** (n = 1)	**IB1** (n = 20)	**IB2- IVB** (n = 16)
Strict follow up (n = 34)	2 (66.7%)	1 (100%)	17 (85.0%)	14 (87.5%) *
Conization (n = 3)	1 (33.3%)	-	2 (10.0%)	-
Trachelectomy (n = 1)	-	-	1 (5.0%)	-
NACT (n = 2)	-	-	-	2 (12.5%)

Data are presented as n (%).
NACT; neoadjuvant chemotherapy.
* Three patients died of disease. Case #1 (Stage IB2, Adenocarcinoma): diagnosed at 36 gestational weeks, delivered at 37 gestational weeks, and died at 42 months postpartum. Case #2 (Stage IIA2, Squamous cell carcinoma) was diagnosed at 27 gestational weeks, delivered at 27 gestational weeks, and died at 33 months postpartum. Case #3 (Stage IVB, Adenocarcinoma) was diagnosed at 37 gestational weeks, delivered at 37 gestational weeks, and died at 24 months postpartum.
